# Sustained ethical analysis of global dilemmas and country-level decision making during and post the COVID-19 pandemic: A systematic review

**DOI:** 10.12669/pjms.38.4.4755

**Published:** 2022

**Authors:** Azza Sarfraz, Zouina Sarfraz, Mohammad Ashraf, Huma Ashraf

**Affiliations:** 1 Azza Sarfraz, Aga Khan University, Karachi, Pakistan; 2 Zouina Sarfraz, Fatima Jinnah Medical University, Lahore, Pakistan; 3 Mohammad Ashraf, Wolfson School of Medicine, University of Glasgow, United Kingdom; 4 Huma Ashraf CMH Lahore Medical College, Lahore, Pakistan

**Keywords:** Allocation of Health Care Resources, Clinical Ethics, Decision-making, International Affairs, Public Health Ethics, WHO, Pakistan

## Abstract

The COVID-19 pandemic has highlighted the vulnerability of countries worldwide and their abilities to cope with the fast-paced demands of the research and medical community. A key to promoting ethical decision-making frameworks is by calibrating the sustainability at regional, national, and global levels to incorporate coordinated reforms. We performed a sustained ethical analysis and critically reviewed evidence addressing country-level responses to practices during the COVID-19 pandemic using PubMed (MEDLINE), Scopus, and CINAHL. The World Health Organization’s ethical framework proposed for the entire population during the pandemic was applied to thematically delineate findings under equality, best outcomes (utility), prioritizing the worst off, and prioritizing those tasked with helping others. The findings demarcate ethical concerns about the validity of drug and vaccine trials in developing and developed countries, hints of unjust healthcare organizational policies, lack of equal allocation of pertinent resources, miscalculated allocation of resources to essential workers and stratified populations.

## INTRODUCTION

The COVID-19 pandemic exposed the vulnerability of countries globally and their ability to cope with the ensuing responses fast-paced demands of the research and medical community.^1^ Pakistani and international health care systems are under obligation to provide sufficient access to healthcare to all.^2^ However, the burden on health care systems has rendered the rationing of resources to make dire choices.^3^ The catastrophic impact of the pandemic has highlighted the discrepancy in contextual circumstances and ensuing limitations in access to hospitals, ventilators, vaccines, and pharmacological therapy for all. The overwhelming burden to health care systems and the ensuing unequal distribution of certain key resources to high-income countries (HICs) has rendered the already-scarce capacity of resources insufficient to meet everyone’s needs.^4^ To address ethical concerns, the concept of sustainability takes the forefront that promotes the efficient management of resources. The COVID-19 pandemic is synonymous with incidences of human tendencies to act according to convenience, expediency, and self-interest, known as unsustainable rapacity. A major public health challenge is the ability to tackle and identify ethical challenges posed by the ongoing COVID-19 pandemic, particularly within the contextual settings.

The contextual considerations are perspective given different principles and values are determining ethical considerations. COVID-19 vaccines, once ready for public use, given to high-risk populations such as health care workers, and the elderly are justified. However, the allocation of a scarce protective resource may be distributed randomly within a high-risk subgroup if the assumption supports equal benefits within the predetermined sub-group. The ethical considerations for a case-by-case basis in the use of another limited protective resource might differ such as the use of ventilators in the COVID-19 pandemic. Practical applications of ethical analysis and recommendations by the BC COVID-19 Ethical Decision-Making Framework (EDMF): Interim guidance is tied in sustainable responses at regional, national, and global scales to incorporate coordinated reforms.^5^ At the core, public health ethics incorporate respect, the harm principle, fairness, consistency, and least coercive and restrictive means, working together, reciprocity, proportionality, flexibility, and procedural justice. While fair ethical practices consider transparency, inclusiveness, consistency, they may not gain the trust and solidarity of public health due to its inherent nature. Given the global impact of COVID-19, the responsibility to share resources such as vaccines may be at risk of resource ownership out of self-interest and prioritization. However, priority sub-groups determined by ethical principles dictate that higher-risk settings and populations are to be prioritized.

## METHODS

To address the gaps in the literature, a systematic-sustained ethical analysis was deemed appropriate to synthesize and integrate relevant literature with a lack of comprehensive understanding of the research questions. The review aimed to recognize the extent, burden, and gaps in the existing literature to guide future research and practices. The following keywords were used: COVID AND ethic*. We included any study addressing ethics during COVID-19, and did not omit studies if they were qualitative. Studies published in PubMed (MEDLINE), Scopus, and CINAHL full text from January 1, 2020, until December 31, 2020, were included. Articles focused on public health and ethical frameworks for analysis were considered relevant for our review with special consideration given to literature catering to low and middle-income countries. The framework proposed by the World Health Organization is tied in with those who require priority access to scarce resources amid the pandemic.^6^ All reviewed studies were analyzed using a single framework that was tabulated and the findings were presented in the results.

### Equality:

In principle, every person’s interest concerning healthcare services or resource allocation is pertinent during the COVID-19 or any other disease outbreak, unless there are reasons to justify a differential prioritization of existing or potential resources. Equality may be the most suited guide to allocating scarce resources among populations or individuals who may have the same benefit from the given resource. For example, ventilators among those patients who have similar clinical indications for yielding benefit.

### Best Outcomes (Utility):

The principle is used to reason with the allocation of resources based on their capacity and to maximize the most benefit while reducing the harm. For example, resources may be used to save more lives at that time-point. This may be the most appropriate guide to allocating resources to recipients that will have different benefits among different individuals. For example, ventilators will be expected to derive maximum benefit among patients who are relatively healthier as opposed to those that are more than likely to outweigh all benefits.

### Prioritizing the Worst Off:

The principle has ambivalent views. In our review, we will attempt to justify the allocation of resources to those that have the highest medical need or those who are the highest risk. It is an appropriate guide to allocate resources and protect those at risk. For example, the provision of personal protective equipment (PPE) for healthcare workers or those who most require the provision of drugs given a short supply for those who require them most urgently.

### Prioritizing Those Tasked with Helping Others:

The principle is used to ensure that resources are allocated to those that have certain talents or skills and who have talents to save others, or because resources are owed to them due to them participating in helping others. This is the most imperative principle to guide the allocation of resources to first responders or health care workers.

The review analyzed the ethical concerns and challenges identified in the literature to identify the ethical practices pertinent to the COVID-19 response. A search of academic literature was conducted to address the following questions:

(1) How have countries incorporated ethical practices in their COVID-19 pandemic response?
(2) What is to be learned for the COVID-19 pandemic as well as future epidemics and pandemics?

## RESULTS

Three databases including PubMed, Scopus, and CINAHL full text were searched with a total of 2,124 results. We identified 15 papers for review including around less than half (n=7) studies reported on the country-specific ethical practices concerning the validity of conducting drug and vaccine trials in developing countries, and government-wide ethically just policies (Table-I). Over half (n=8) addressed the lessons learned during the COVID-19 pandemic and applicability to future pandemics and epidemics (Table-II). The majority of the records were excluded as they were irrelevant to our predetermined research questions.

**Table-I:** Application of the WHO framework to countries’ response during the COVID-19 pandemic.

Author (s)	Study type	Equality	Best outcomes (utility)	Prioritizing the worst off	Prioritize those tasked with helping others	Implications for practice
Macklin^7^	Essay	Mexican government (CSG) used age-based criteria for the allocation of scarce resources conflicting with the “equal worth of life” principle in its first version.	“Save the most lives” principle was promoted by the guidelines in the final version.	In the final version, the "save the most lives" principle instead of age-based criteria allows the healthcare workers to triage patients effectively.	Priority to healthcare workers involved in COVID-19 care is endorsed in live-saving emergencies by the guidelines.	Health-care workers can use the “luck by draw” method to triage the patients.
Gebbiaa^8^	Essay	Italian Government reduced the medical litigation to fraud only. This amendment without extensive guidelines may pose risk to the implementation of all ethical principles including equality.	Reduced litigation may allow the healthcare force to focus their time and effort towards serving the public.	Postponement of non-urgent treatments shifts resources towards emergent cases.	The law addressed the need to mitigate the psychological and financial distress of healthcare workers in the current crises.	With reduced litigation, healthcare workers can maximize their efforts towards emergent COVID-19 patients.
Singh^9^	Perspective	The marginalization of Africa from the COVID-19 vaccine trials contradicts the principle.	Vaccines may not work for Africans due to differences in immunity among populations that can deprive Africans of an efficacious vaccine.	WHO excluded Africa to prevent the vulnerable population from exploitation during the vaccine trials.	The exclusion of the entire population disfavors everyone, including people actively helping others in Africa.	African health sector may have to face an extra challenge if the vaccine fails in the African population. Global efforts are required to ensure the safe implementation of trials in Africa without exploitation.
Heidi J Larson^10^	Worldview	Iranian authorities warned healthcare workers regarding the sharing of information about scarce resources and infected people. This may hinder the equitable distribution of resources as it interferes with transparency and may advantage those in power.	False claims, “all controlled” by US and Canadian authorities delay effective measures to control the pandemic which may further increase the infection rates. It may benefit economic interests; however, it compromises the health of most people.	Improper information handling led to the spread of fabricated health recommendations which poses safety risks for the public.	Healthcare workers under-reported cases due to fear of their practice being shut down. It puts physicians under financial strain and increases safety risk for themselves and patients.	Physicians need to ensure proper dissemination of medical facts while clarifying the misinformation.
Bakewell et al 11	Commentary	Canadian law mandated duty of care for an already established doctor-patient relationship. It may marginalize COVID-19 patients from care as physicians may choose not to establish relationships with these patients.	Canadian Medical Protective association urges physicians to provide care at the highest possible standards, i.e. to use resources in the best possible way.	Doctors may opt-out of direct care if they have personal characteristics like comorbidities and age that may increase the risk of harm to them and can choose to participate in indirect patient care instead,	Priority is given to front-line workers for resources and equipment due to the additional risk they face.	Doctors can weigh the risk of Covid-19 for themselves to determine whether they can provide patient care directly or indirectly.
Shadmi et al.^12^	Commentary	Australia has a “health for all” care system but it excludes temporary residents.	Doctors are motivated to adjust the cost of vulnerable patients through financial incentives, increasing the utility of the workforce for the patients.	Lack of access to telehealth due to poor internet availability among less privileged may lead to a major health risk.	Financial preference for the health care sector e.g. payment of 1 billion $ for COVID-19 response.	The Healthcare workforce needs to outreach the patients with less accessibility to telehealth to ensure the inclusion of all individuals.
Garg et al.^13^	Viewpoint	Digital contact tracing via phones leads to inequitable surveillance, excluding digitally illiterate old population and remote areas with poor access to telecommunication.	Digital surveillance increases surveillance diameter compared to previous methods of surveillance	Digital surveillance is a threat to an individual’s privacy.	Proper surveillance allows controlling the spread of COVID-19, improving outcomes for vulnerable health care professionals.	Proper dissemination of data to ensure its usage only to control the spread of COVID-19.

**Table-II:** Ethical applications based on lessons learned from the COVID-19 pandemic.

Name of Study	Type	Equality	Best Outcome	Prioritize the worst off	Prioritize those tasked with helping others	Implications of future practice
Gostin et al.^14^	Essay	The author sub stratifies hospitalized patients into two groups. COVID19 and other. Equality cannot guide decision making due to a shift towards public health ethics.	Public health has to be prioritized and within it those who are to benefit the most. Emphasis is on prevention, ensuring an overall best outcome is achieved.	Divert resources towards the epicenter of the pandemic and ensure social justice by prioritizing nursing homes and other proximity areas.	Give industry-government support to produce resources required by health care professionals in mass numbers. Priorities sick health care workers as without them all other groups suffer.	Public health-centered care requires a massive change in the decision-making process, resource allocation, and prioritization of patients.
Angeloss^15^	Editorial/Essay	Equality is not possible due to shifting in public health ethics rather than patient-centered ethics.	In surgery, elective cases must be canceled when appropriate. Often these patients are those who have the best outcomes.	Only operate when necessary for example on emergency and emergency elective cases.	Protocols must be established that take in to account the risk to the surgeon. Priorities their health as they may even need to work with the care of COVID patients in an area where the staff is inadequate.	Future surgeons must have a firm understanding of principles of infectious disease and protocols must be developed at departmental and national levels for future pandemics.
Prachand et al.^16^	Original article	NA	The MeNTS score provides a framework to decide who should be operated on. It prioritizes those who are worst off and within those, those who have the best outcome. It takes into account the risk to the surgeon as well.	The MeNTS score provides a framework to decide who should be operated on. It prioritizes those who are worst off and within those, those who have the best outcome. It takes into account the risk to the surgeon as well.	The MeNTS score provides a framework to decide who should be operated on. It prioritizes those who are worst off and within those, those who have the best outcome. It takes into account the risk to the surgeon as well.	A similar framework should be developed for surgical subspecialties.
Shuman et al.^17^	Editorial	NA	NA	The conflict between caring for cancer patients as oncology staff may be facing shortages of nurse’s doctors who may be staying off for their protection. The solution is to employ E-visits as this would minimize risk to both patient and doctor.	The conflict between caring for cancer patients as oncology staff may be facing shortages of nurse’s doctors for their protection. The solution is to employ E-visits	Introduce E-Visits when necessary and only allow relevant staff on the wards.
Shuman et al.^18^	‘Special Issue’/Essay	Equality cannot guide decision making as multiple complex factors interfere here however the authors have suggested a way to mitigate this.	Provide surgery to those who would not require ICU beds post-op. This includes reconstructive work. Shift treatment options to medical from surgical where applicable	Prioritizing the worst off by developing a system which recognizes those who can either wait for surgery or be accommodated by medical management	The authors here clearly state that the duty to protect oneself and others from harm is above the duty of a doctor to their patients.	Institutions should share treatment paradigms if/when possible to prevent public distrust about the quality of care being received. Consult the marginalized and minimize intrinsic/extrinsic bias in decision making.
Turale et al.^19^	Collaborative Editorial	Equally all nurses regardless of their location of work should have equal access to mental health care	NA	COVID 19 adversely affects the poor and thus those with comorbidities and the disenfranchised. There is a call to focus on this cohort of patients.	Call to provide mental health care for all nurses. Nurses’ well-being must be put above all else and doctors should not be promoted to have a martyr mentality.	Have a strong support network for healthcare workers which would allow them to easily transition out of future pandemics to regular practice.
Dawson et al.^20^	Original Article	Equality is impractical as a person’s health status is often reflected in their socio-economic determinants thus preventing discrimination is difficult. The young should be given priority over the old.	Prioritize the young as they are likely to have the best outcome.	Authors argue that healthcare should get the most out of their limited resources. This echo’s the principle of the best outcome. They also state this would be cost-effective.	Priority should be given to doctors so that they may be able to save those with the best outcomes.	Decisions should be patient-specific but should broadly lead to the most use of the resource itself and this will address the issue of ’value of money’. In addition to this, it would lead to benefit of those with the best outcomes.
Emanuel et al.^21^	Editorial	First come first serve is excluded as it is not appropriate in a pandemic but a random selection/lottery method may be used amongst people with a similar prognosis	The principle of sickest first/youngest first should only be used when it aligns with maximizing benefits.	The authors state that ICU beds and ventilators should be given to those who are expected to benefit the most from them.	Promote instrumental value by giving priority to those who can save others or who have done so in the past	Maximize the value of resources in a pandemic. Create an incentive for finding optimal treatment by giving preference research participants. This principle should apply after principles of the best outcome.

To our understanding this is the first study to analyse ethical practices in a sustained and analytical manner during the era of COVID-19, particularly with implications for Pakistan. Studies show ethical concerns about the validity of drug and vaccine trials in developing and developed countries, hints of unjust healthcare organizational policies, the lack of equal allocation of pertinent resources, miscalculated allocation of resources to essential workers and stratified populations.

## DISCUSSION

### The global response to ethical practices during the COVID-19 pandemic:

The role of governments in managing the pandemic is critical. Guidelines for the ethical practice of healthcare need to evolve as facts about COVID-19 progression and spread unfold. Consejo de Salubridad General (CSG) of the Government of Mexico posted two versions of guidelines regarding the allocation of health resources. Both versions promoted the distribution of resources based on equity i.e. free from the influence of race, religion, political affiliation, social value, nationality, gender, or race. The first version used age-based criterion derived from the “complete lives” principle based on justice law to triage patients. The criterion was highly criticized as physiological age is not equivalent to chronological age and it undermines the principle of “equal worth of life”. Therefore, the guidelines were revised to include “luck of draw” (by chance) to triage patients with an exception of priority given to healthcare workers involved in the management of COVID-19.^7^ The Mexican government showed adaptation to the changing nature of the pandemic by improving its guidelines in the second version regarding triage.

An additional challenge to Pakistani doctors and the public is presented by the practice of non-validated telehealth models for patient care. The safety and efficacy of telehealth models have not been established which could lead to a major ethical risk for the healthcare practice based on mitigating damage to patients. The non-validated model increases the risk of litigation for doctors by patients which may result in excessive legal trials leading to decreased efficacy of healthcare and increased economic burden leading to low utilization of resources towards the pandemic. To address the problem, the Italian Government Cabinet reduced the litigation of doctors to “fraud cases” only to meet the demand for extraordinary emergencies. The law implies that healthcare professionals may not be held accountable for involuntary violation of guidelines for COVID-19 emergencies but is expected to follow guidelines in concrete situations. It may allow the continuance of patient care while maximizing the current use of resources towards emergencies rather than litigation charges.^8^ However, reduced litigation can lead to malpractice, it is important to assess the implications caused by the laws to formulate extensive and clearer frameworks for future health care challenges.

Surveillance is an important part of the Covid-19 response to governing the increasing cases. The Indian government established a surveillance program, Aarogya Setu (“A bridge of health”) which uses mobile location for contact tracing. The surveillance method traces contacts via the location data on the phone of the confirmed COVID-19 case and informs contacts in the proximity of the phone.^13^ Technology has provided great solutions to growing problems, but it also leads to major ethical implications. As per the policy, reporting of COVID-19 to the government is optional for the diagnosed patient. It respects patients’ autonomy but decreases the accuracy of reporting which increases public health risk against highly infectious diseases. Digital illiteracy among children and the old population also adds to the underreporting of COVID-19.^13^ Indian government addressed a major issue via digital surveillance but it is a major threat to the patient’s privacy. Extensive measures need to be devised to ensure safe usage of data only for the control of pandemic.

Given the ethical implications of COVID-19, it is important to create valid policies to ensure equitable resource distribution, which requires transparency of information among governing bodies and the public.^9^ The statements passed by various authorities like “all controlled” to calm the public falsely can result in exacerbation of an inflamed situation that disseminates distrust among the masses leading to further chaos. It is important to communicate the truth clearly to have organized management of an ongoing crisis as misinformation poses a major safety risk. A clear example of the ethical framework of decision-making is provided by the Canadian Medical Association, which acknowledges the duty of both healthcare workers towards the public and vice versa in handling the pandemic. It speaks to the maximum utilization of resources. Canadian Association of Emergency Physicians (CAEP) suggests physicians with high-risk characteristics for COVID-19 like comorbidities to engage in indirect rather than direct patient care, mitigating the risk for those helping others.^11^ While Canadian Medical Protective Association recognizes the legal duty doctors to continue patient care at set standards, Canadian Governments’ reciprocal efforts including the provision of resources like PPE ensures safe management of Covid-19 for doctors and patients.^11^

The WHO’s policy, “Health for All’’ is adopted by most countries. The Covid-19 pandemic has revealed the loopholes in the ethical practice based on equities e.g. Medicare in Australia does not cover the marginalized refugees and immigrants. In the USA, minorities showed higher rates of COVID-19 infection e.g. African Americans accounted for 50% of all COVID-19 cases in Chicago.^22^ For testing, major insurance companies have waived co-pays. However, minorities have poor insurance rates, and most do not have a prior established doctor-patient which can exclude these populations from current vaccine trials.^12^ Lack of access to clinical trials among the minorities may lead to decreased efficacy of vaccines as immune response varies among ethnicities. Global efforts are needed to conduct vaccine trials based on ethical principles that protect the population from malpractice, for the current and future global outbreaks.

### Lessons learned from the COVID-19 pandemic in Pakistan and applicability to future outbreaks:

**Fig.1:**
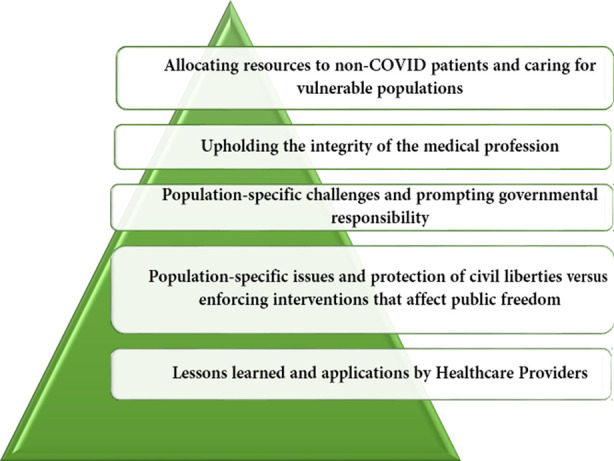
Key ethical applications for the Pakistani population.

To better prepare for pandemics in the future relevant professional bodies in Pakistan representing specialties should prepare guidelines to aid in the management of everything patients to resources. These include guidelines for health care professionals and the individual level and national bodies, unions, and particularly pharmaceuticals due to their influence on health care in high-income countries. Particularly, Pakistan will need guidelines that address challenges that are unique to them and these include far less equipment, staffing shortages, and the probable reality of being the last group to receive vaccines. Fig.1 represents key ethical concerns during and post the COVID-19 pandemic in Pakistan, which are applicable across developing countries worldwide.

The pandemic has taught the Pakistani population that a lack of harmony and a non-uniform approach may lead to distrust from patients as multiple treatment paradigms. It is pertinent to promote a single reliable source to provide information to the public.^23^ Professional bodies representing their specialty must make recommendations for consistency across hospitals which must be evidence-based consensus and for this to occur there must be a collaboration between institutions to maintain uniformity in treatment paradigms as much as possible.^24^,^25^ As Gostin et al. suggest, resource limited countries like Pakistan may struggle to promote equality, a guiding principle that focuses on ’public health care’ as opposed to the more common ’patient-centered care’.^26^,^27^ In the future, the Pakistani government should give adequate support and mobilize industry to ramp up the production of necessary equipment and relay to the healthcare centers most in need. The pandemic has disproportionately affected the socioeconomically and culturally stratified populations in Pakistan particularly women from marginal communities to whom extra financial support should be targeted towards. An important point the COVID-19 pandemic has raised is the enforcement of social distancing vs protection of civil liberties and disenfranchised ethical and cultural minorities in Pakistan. For the ethical wellness of Pakistan in the future, it is important to foster trust with such communities to gain their corporation. It is clear that while a tragedy, in the setting of pandemics, equality is not a leading principle in Pakistani healthcare systems. Consensus has been reached that in the context of a pandemic maximizing benefit will be the most important aspect.^21^ COVID 19, personal protective equipment, testing, vaccines, ICU beds and ventilators ought to be first given to health care front line staff without whom the critical infrastructure would collapse.^28^ For the future, Pakistan ought to partake in international collaborative registries so that data is shared and trials can be commenced on large scale immediately.

## CONCLUSION

This sustained ethical analysis and systematic review suggests that although far from over, the pandemic has created deep divides in the Pakistani and international societies. The COVID-19 pandemic continues to provide crucial lessons that we must pay heed to if we are to grow a crisis with overarching effect systems and structure. While pandemics tend to burden health care services, they have prompted clinicians to shift from ’patient-centered’ ethics to ’public health’ ethics, the later a concept that few first-world and Pakistani health care professionals are familiar with.

### Authors’ Contributions:

**AS and ZS** conceived, designed, did the data collection, writing and editing of manuscript.

**MA and HA** did the data collection, manuscript writing, and editing of the manuscript.

**ZS** is the guarantor and is accountable for all aspects of the work in ensuring that questions related to the accuracy or integrity of any part of the work are appropriately reported.
